# Physical and Sedentary Activities and Childhood Overweight/Obesity: A Cross-Sectional Study among First-Year Children of Primary Schools in Modena, Italy

**DOI:** 10.3390/ijerph18063221

**Published:** 2021-03-20

**Authors:** Stefania Paduano, Antonella Greco, Lucia Borsari, Chiara Salvia, Stefano Tancredi, Jenny Pinca, Simona Midili, Alberto Tripodi, Paola Borella, Isabella Marchesi

**Affiliations:** 1 Department of Biomedical, Metabolic and Neural Sciences, Section of Public Health, University of Modena and Reggio Emilia, 41125 Modena, Italy; stefanotancredi@gmail.com (S.T.); paola.borella@unimore.it (P.B.); isabella.marchesi@unimore.it (I.M.); 2 Department of Public Health, AUSL Modena, 41126 Modena, Italy; an.greco@ausl.mo.it (A.G.); l.borsari@ausl.mo.it (L.B.); 3 Primary Care Health Authority and Services, AUSL Modena, 41124 Modena, Italy; chiara.salvia13@gmail.com; 4 Department of Public Health, Food Hygiene and Nutrition Service, AUSL Modena, 41126 Modena, Italy; j.pinca@ausl.mo.it (J.P.); s.midili@ausl.mo.it (S.M.); a.tripodi@ausl.mo.it (A.T.)

**Keywords:** physical activity, sedentary behaviours, childhood overweight/obesity, parental education, lifestyle

## Abstract

Children obesity is a serious public health issue. This study aimed to investigate physical/sedentary activities of first-year primary schools children in Modena, and their association with overweight/obesity and dietary habits of children and family characteristics to identify the risk factors for unhealthy lifestyles. Child physical/sedentary activities were gathered through an anonymous questionnaire administered to parents, as well as family characteristics and weight/height of child and parents. Logistic regression models, eventually adjusted for parents’ sociodemographic characteristics, were used to analyze data. Questionnaires were delivered by 660 families (74.2%), of which 72 without anthropometric data were excluded. Three out of four children spent in physical activities less than 7 h/week, while 63.9% dedicated to sedentary activities two or more hours/day. From multivariate analysis, the habit significantly affecting children’s overweight/obesity was spending time on tablets/Personal Computers/mobile phones/videogames. Higher parental education level resulted in a protective factor for implementing unhealthy lifestyles in terms of time dedicated to physical/sedentary activities. Our results suggest the need of interventions to increase time for physical activity and to promote a responsible use of digital media involving the entire families to reach all parents regardless of their education and nationality with a possible relapse on other family members.

## 1. Introduction

Worldwide, childhood obesity has increased 10 times in the last 40 years, and it is considered a serious public health issue [1,2,3]. In the 19 countries that participated in the European Childhood Obesity Surveillance Initiative (COSI), the prevalence of overweight children among 6–9-year-old age group ranged 18–52% in boys and 13–43% in girls and that of obesity ranged 6–28% among boys and 4–20% among girls [4]; in many countries 1 out of 4 obese children are severely obese [5]. Some physical and psychosocial complications are already present in obese children and tend to worsen in adulthood. Indeed, obese children are likely to become obese adult and are at risk of various chronic diseases [1,6,7]. Therefore, childhood obesity prevention is a priority for public health policies.

In Italy, a surveillance system named “Okkio alla Salute” was created in 2007 with the aim to evaluate childhood overweight/obesity trend among 8–9-year-old children and the associated modifiable behaviors [8]. The 2019 data show that the prevalence of overweight children in primary school is still 20.4% and obese is 9.4% [8].

Being overweight in school-age children is a condition associated to several determinants. Living in an obesogenic environment promotes high caloric consumption, such as increasing use of sugar-sweetened beverages and sweet snacks, poor physical activity and excessive use of screens [9,10,11]. Aside from eating habits, total screen time, e.g., television or computer time, is related to childhood overweight and obesity, some studies demonstrated that time dedicated to physical activity does not compensate the adverse effects of screen time [12,13,14]. The TV in children’s bedrooms has been related to overweight risk [9]. In addition, several studies have shown that excessive screen time negatively affects children’s dietary habits, leading mostly to poor nutrition food choices and a lower consumption of fibre and fruit [15]. Moreover, TV advertisement can favorite an increase in food intake [14,15,16]. 

Therefore, it is fundamental that children achieve a balance between calories intake and energy expenditure by appropriate dietary intake, physical activity and reduced total screen time. These changes should affect not only the child, but also the whole family. For 6 year-old children, WHO suggests at least 60 min of moderate-to-vigorous physical activity per day including strengthen muscle and bone exercises three times per week [17,18]. The Italian Society of Pediatrics and Italian Society of Pediatric Endocrinology and Diabetology recommend to limit screen time to 2 h a day or less [19].

A systematic literature review shows that in Europe less than 50% of children and adolescents comply with physical activity recommendations, regardless of methods of measurement, with great variability among countries [20]. Italy is among the nations with less adherence to these guidelines, with only 13% of 11 years-old children adhering to such guidelines [20]. An increase of sedentary behaviors and a decline of physical activity is recorded between 6 and 11 years of age [21]. The HBSC (Health Behavior in School-aged Children) study demonstrated that this trend continues also during adolescence: a decline of moderate-to-vigorous physical activity was observed with an increase of screen activities such as watching television and using other devices [22].

For a long time in Modena, Northern Italy, a community-based health initiative has been active in promoting healthy lifestyles amongst children attending primary schools [23,24,25]. Within this project, we conducted a study through an anonymous *ad hoc* questionnaire administered to the parents to investigate the lifestyles among children attending the first year of primary school. The main aims of this study were to investigate time dedicated to physical and sedentary activities and its association with overweight/obesity and dietary habits of children and to identify the family characteristics influencing children lifestyles.

## 2. Materials and Methods

### 2.1. Study Design

In 2018, a cross sectional study was conducted among first-year children (aged 6–7 years) attending the state primary schools in Modena Municipality. The schools’ participation in the initiative was on voluntary basis. Fifteen out of 27 primary schools, for a total of 890 children attending 38 first-year classes participated to the study without any exclusion criteria. 

### 2.2. Data Collection

Parents were required to fill out a self-administered questionnaire, accompanied by a participant information sheet and a consent form. The participation was on voluntary basis and data were collected anonymously. The Emilia Ethics Committee (called “Area vasta Emilia Nord”), our reference for on field studies, was consulted for the approval of the study. It allowed the start-up, since the returned questionnaires anonymous, and delivered in a sealed envelope, without any reference to the school and the compiler as well. 

The questionnaire consisted of three parts. The first part was about the socio-demographic characteristics of each child (sex, month and year of birth,) and their parents (year/place of birth, education level, nationality), the anthropometric measurements (weight and height) of both children and parents. The second section collected the children’s eating habits, such as daily consumption of breakfast and mid-morning snack, fruits and vegetables, carbonated or sugary drinks. The third part gathered data on the children’s lifestyles: days per week (at least one hour/day) spent in organized or non-organized physical activities, hours per week spent in all physical activities (sports + games) and hours per day spent in sedentary activities (watching TV, gaming on tablets, PCs (Personal Computers) and mobile phones) and if there is a TV in the child’s bedroom. In addition, information was collected about how children go to school (walk, bicycle, car or bus). From 2020 WHO guideline, sports cover a range of activities performed within a set of rules and undertaken as part of leisure or competition [18]. Sporting activities involve physical activity carried out by teams or individuals and may be supported by an institutional framework, such as a sporting agency. Games consist of non-organized recreational physical activities, which are therefore not included in sports.

### 2.3. Data Analysis

Participants without child anthropometric data were excluded, as weight and height were used to calculate the body mass index (BMI = weight (kg) ÷ height (m)^2^) used for determining clinical weight category for each child in accordance with BMI cut-offs of the International Obesity Task Force (IOTF) [26]. The children were classified as underweight, normal weight, overweight and obese. The parental BMI categories were underweight (BMI < 18.5 kg/m^2^), normal weight (18.5–24.9 kg/m^2^), overweight (BMI 25.0–29.9 kg/m^2^) and obese (BMI ≥ 30 kg/m^2^) according to the Forbes definition [27]. The participants were divided into two groups, namely under/normal weight and overweight/obese.

The differences in physical/sedentary activities between males and females were evaluated using χ² test. The influence of family’ characteristics on child’s incorrect habits (<3 and <2 days per week (at least one hour/day) spent in organized activities (sports), <3 days per week spent in non-organized activities (games), <7 h per week in physical activities (sports + games), car/bus to go to school, ≥2 h daily spent in tablet/PC/mobile phone use or in TV viewing or in all sedentary activities, TV in child’s bedroom) was analyzed using univariate regression models in order to compute the odds ratio (OR) with 95% confidential intervals (95% CI). Moreover, the influence of children’s unhealthy behaviors on their weight status was assessed using univariate and multivariate logistic regression models, adjusted for parents’ characteristics (nationality and education). Finally, the association between incorrect dietary habits (skipping breakfast, skipping mid-morning snack, fruit/vegetable consumption <once a day, carbonated drinks consumption ≥once a day and sugary drinks consumption ≥once a day) and physical/sedentary activities was evaluated using univariate regression models.

The data collected were analyzed using STATA Software (release 15, StataCorp, College Station, TX, USA), with the level of significance set at *p* < 0.05.

## 3. Results

A total of 660 (74.2%) families delivered the questionnaires, and after excluding those without anthropometric data, 588 children were included in the study, 313 (53.2%) males and 275 (46.8%) females. The missing values were less than 5% for each item, except for paternal nationality (6%) and paternal self-reported weight status (15%). The characteristics of the study population are reported in Table S1. According to the weight categories defined by the IOTF [26], 8.1% of children were underweight, 66.7% normal weight, 15.0% overweight and 10.2% obese.

Table 1 shows the physical activities and sedentary behaviors of the children according to the child’s sex (Figure S1). Among children 51.5% dedicated ≥2 days per week performing organized physical activities and 15.1% spent ≥ 3 days per week with percentages significantly higher among males than among females.

Overall 27.8% of children did not perform any sport, without difference by sex. No significant difference between males and females was observed for non-organized physical activities and ways to go to school. A total of 450 children out of 588 (76.5%) spent less than 7 h weekly in physical activities. Among 376 children (63.9%) who spent ≥2 h per day in sedentary activities with a higher percentage of boys than girls.

The results of univariate logistic regression models applied to physical activities are reported in Table 2. Boys were more likely to dedicated time weekly (≥3 or 2 days per week) to organized physical activities than girls, whereas children of foreign parents were less likely to perform these activities. Performing organized physical activities was positively associated with higher levels of parental education.

Table 3 presents the association between some family characteristics and sedentary behaviors. Boys were more likely to dedicate two or more hours daily to sedentary activities. Children born to foreign fathers as well as those with overweight/obese fathers spent ≥2 h per day in tablet/PC/mobile phone use and watching TV. Dedicating less than 2 h daily to sedentary activities was positively associated with higher levels of parental education.

The influence of children lifestyle on childhood overweight/obesity prevalence was analyzed using various logistic regression models (Table 4). The results of univariate models showed that spending two or more hours daily in tablet/PC/mobile phone use or in overall sedentary activities was significantly associated with childhood overweight/obesity, whereas only the first factor remained significant in the multivariate analysis, regardless of the presence of a television in the child’s bedroom.

Moreover, children who spent ≥2 h per day in sedentary activities were more likely to engage in unhealthy dietary habits (Table 5). In particular, tablet/PC/mobile phone use and watching TV were associated with inadequate fruits and vegetables consumption (< once a day) and drinking sugary beverages daily. In addition, tablet/PC/mobile phone use was related to skipping breakfast and consuming carbonated drinks. Children who had TV in their bedroom were more likely to skip breakfast and drinking carbonated and sugary beverages.

## 4. Discussion

This study conducted among a representative number of 6–7 year-old children living in Modena (Italy) shows an insufficient time spent in physical activity and an excessive screen time, favoring overweight/obesity. Three out of four children spent in physical activities less than 7 h weekly, while 63.9% dedicated to sedentary activities two or more hours daily. Interestingly, the most relevant risk factor for childhood overweight/obesity was the excessive screen time, whereas higher level of parental education resulted in a protective factor for implementing unhealthy lifestyles in terms of time dedicated to physical and sedentary activities. Noteworthy is the fact that children who spent more time in sedentary activities were also more likely to adopt unhealthy dietary habits.

As the imbalance between energy intake/expenditure is the most significant risk factor of overweight / obesity in children, in our study we analyzed how much time children spent in movement and how much time they spent in screen time (TV, computers, tablets, videogames, mobile phones), and their association with family characteristics, children overweight/obesity and some eating habits.

Although National and International Guidelines recommend at least 60 min of moderate to vigorous physical activity every day [17,18,19], a 2010 review reports that there is no evidence about a greater benefit of one hour daily compared to a total of 7 h weekly with some days of non-activity [28]. For this reason, we considered as active children who had a total of at least 7 h weekly spent in physical activities, organized or not. In this study, it was observed that only 23.5% of children performed physical activities for seven or more hours weekly with no difference based on sex or weight status.

Nevertheless, a higher males’ percentage engaged in organized physical activities two or more days weekly for at least one hour daily compared to females as also reported by Verloigne et al. [29]. It was observed that about 28% of our study group did not perform any sports, a higher percentage compared to those (17%) detected in 2016 during the National study “OKkio alla Salute” carried out on 8–9 year-old children in our region (Emilia-Romagna). They found that 13.9% of males and 18.8% of females not involved in organized physical activities [30]. As children from six to eleven years tend to decrease the time dedicated to moderate to vigorous physical activity [21,31], our study suggests a worsening. Children born to highly educated parents tended to spend more days per week (≥3 days / week) in organized physical activities: having at least one parent with a university degree had a positive influence on sports practice. Children with at least one foreign parent were less likely to practice structured physical activity. This data could be related to limited resources of parents or to their lack of experience in organized sports [32]. In addition, Renzaho et al. reported that for foreign parents, large body size was associated with being beautiful and wealthy, whereas slimness with chronic illness and poverty. For this reason, they adopted strategies for weight gain in children including tailored food habits and restrictions on children’s physical activity [33]. 

No association was found between the physical activity and the weight status of the children. Although the questionnaire is not the ideal tool to detect the children activities, our result is in line with findings reported by Wijtzes et al. [34]. Indeed, they have shown that the practice of a physical activity was inversely correlated with fat mass but not with BMI [34]. According to a 2013 review, exercise was effective in reducing the percentage of fat mass in overweight/obese children, but insufficient evidence about an effect in decreasing BMI [35]. Constant exercise probably determined a reduction in fat mass and an increase in muscle mass, so the BMI remains almost unchanged.

Regarding sedentary behaviors, National and International Guidelines recommend limiting the screen time to <2 h a day [19,36]. Considering the total screen time spent for tablet, PC mobile phone use and TV viewing, it emerged that about 64% of children exceed the recommended two hours with a significant difference between boys and girls. Girls appeared to be less at risk of spending two or more hours a day in front of a screen, in line with other studies [37,38], probably due to lower use of videogames than boys. For TV viewing no difference was detected between boys and girls. In our study, about 72% of students did not have TV in their room compared to 56% found nationwide in 2019 [8]. This difference could be related to the children age. In a recent study it emerged that children who had TV in their room increased with growing in their age [37]. As reported by Borghese et al. (2015), children with TV in their room were less likely to comply with the guidelines on screen time probably not only for program TV watching, but also for using TV together with other devices such as videogames [39]. It would be important to eliminate these devices from children bedroom and to favor the use of “exergames” (videogames that require body movement or a physical reaction) considered effective in increasing children’s physical activity and improving their health status [40]. Interestingly, children who has at least one university-educated parent tended to adhere mostly to the guidelines in accordance with other authors [38,39,41]. Moreover, it emerged that the father’s overweight/obesity was associated with screen use for two or more hours daily, as found by Furthner et al. [37]. In our opinion, fathers who did not control their weight status could involve more frequently their children in sedentary activities than the others. 

Of note, the children’s overweight/obesity status is associated with a total screen time of two or more hours a day and in particular with the use of devices other than TV. Indeed, no statistically significant associations was found between TV viewing and overweight/obesity of children according with Alturki et al. [42]. This study conducted in Saudi Arabia showed that time spent watching TV or DVD was not associated with children’s weight, unlike other devices [42]. In line with this notable data, we should consider that the new generations dedicate more time to the use of devices such as PCs/videogames/tablets/mobile phones than that spent watching TV. Fang et al. showed that the increase in the screen time for TV or other device is related to childhood overweight and obesity and this association was more consistent considering only time spent in PC use [14]. 

In our previous studies, we found that skipping breakfast and mid-morning snack and drinking daily carbonated beverages were significantly associated with childhood overweight/obesity, according with other authors [23,43,44,45,46]. Children who dedicated two or more hours a day in sedentary activities were more likely to drink daily sugary beverage and to consume less than once a day fruits and vegetables. In addition, subjects who spent two or more hours a day in front of screens as well as those who have a TV in their room were more at risk of other unhealthy eating behaviors such as skipping breakfast, drinking daily carbonated beverages, in accordance with other authors [15,16]. These studies showed that screen time was associated with the consumption of less healthy foods and with the increase in energy intake [15,16]. The association between overweight/obesity and the use of electronic devices, such as PCs, tablets and mobile phones, can be explained not only by a sedentary lifestyle and by the increase in energy intake during the screens use for unhealthy dietary habits, but also with lower duration and/or quality of sleep hours. According to a Canadian study, the sugary drinks consumption while playing videogames in the four hours before sleep is indirectly related to overweight as these behaviors lead to lower quality of night’s rest [47]. Video exposure causes an alteration in sleep and some experts believe that a short duration of night rest represents a potential risk factor for overweight and obesity in childhood, due to neuroendocrine alterations and consequent increased caloric intake [19,48,49,50].

Health-risk behaviors in childhood may have short-term consequences and also into adulthood long-term consequences [51,52]. For this reason, it is important that these behaviors are modified at a young age. Several factors such as parent’s culture, education and income, family structure, school context and work environment, affect adoption of healthy behaviors from children and their families [23,53,54].

The main strength is the young age of the study population allowed us to assess modifiable risk factors of overweight/obesity in a population that may still benefit from intervention programs. This study has some limitations. The collected data were self-reported from questionnaire, in particular weight and height of both parents and children and the same for time spent in physical activities by children. Moreover, the weight category classification based on BMI value is not able to discern body composition, so it can lead to incorrect classifications. However, our results on weight status are comparable with those measured in national surveillance system and the weight category were defined with the same methods based on BMI in line with national surveillance system [8]. In addition, we assessed one domain of sedentary behaviour in the form of screen time, thus further studies are needed to consider other sedentary activities associated with childhood overweight/obesity.

## 5. Conclusions

Our study has the advantage to include 6–7-years children, documenting that about 30% of them do not practice any organized physical activities and more than 60% spend two or more hours daily in electronic devices use with significant differences between males and females. Of note, the habit significantly affecting children’s overweight/obesity was spending time on tablets/PCs/mobile phones/videogames. Higher parental education level resulted in a protective factor for implementing unhealthy lifestyles in terms of time dedicated to physical/sedentary activities. Therefore, it is important to promote the responsible use of electronic devices by informing families about the consequences of their incorrect utilization.

Further research is needed to investigate the influence of school environment on children lifestyles and the association between children habits and parental perception of child weight status. As children’s lifestyles are affected by their parents’ educational level, nationality and weight status, we suggest the need to devise interventions to promote healthy lifestyles taking into account differences between the sexes and involving the entire families in order to reach all parents regardless of their education and nationality with a possible relapse on other family members. Lastly, these public health interventions could not only improve health status, but also promote social integration with creation of a virtuous circle between school-family and community.

## Figures and Tables

**Table 1:** Sample characteristics by child’s sex.

*Sample Characteristics*	All Children *n* (%) ^a^	Males (*n* = 313)	Females (*n* = 275)	
		*n* (%) ^a^	*n* (%) ^a^	*p*-Value
***Physical activities***				
*Organized activities (sports)*				
*≥3 days per week*	89 (15.1)	56 (17.9)	33 (12.0)	0.047
*<3 days per week*	499 (84.9)	257 (82.1)	242 (88.0)	
*Organized activities (sports)*				
*≥2 days per week*	302 (51.5)	175 (56.1)	127 (46.3)	0.019
*<2 days per week*	284 (48.5)	137 (43.9)	147 (53.7)	
*Non-organized activities (games)*				
*≥3 days per week*	261 (44.4)	144 (46.0)	117 (42.5)	0.399
*<3 days per week*	327 (55.6)	169 (54.0)	158 (57.5)	
*Physical activities (sports + games)*				
*≥7 h per week*	138 (23.5)	82 (26.2)	56 (20.4)	0.096
*<7 h per week*	450 (76.5)	231 (73.8)	219 (79.6)	
*How child go to school by season*				
*winter*				
*bike/walk*	177 (31.2)	92 (30.3)	85 (32.3)	0.598
*motor vehicle (car/bus)*	390 (68.8)	212 (69.7)	178 (67.7)	
*spring/autumn*				
*bike/walk*	291 (51.7)	154 (51.0)	137 (52.5)	0.723
*motor vehicle (car/bus)*	272 (48.3)	148 (49.0)	124 (47.5)	
***Sedentary behaviours***				
*Tablet PC and mobile phone use*				
*<2 h daily*	482 (82.0)	249 (79.5)	233 (84.7)	0.103
*≥2 h daily*	106 (18.0)	64 (20.5)	42 (15.3)	
*TV viewing*				
*<2 h daily*	292 (49.7)	147 (47.0)	145 (52.7)	0.163
*≥2 h daily*	296 (50.3)	166 (53.0)	130 (47.3)	
*Sedentary activities* *(TV + Tablet/PC/mobile phone)*				
*<2 h daily*	212 (36.1)	100 (31.9)	112 (40.7)	0.027
*≥2 h daily*	376 (63.9)	213 (68.1)	163 (59.3)	
*TV in child’s bedroom*				
*no*	415 (72.1)	218 (72.0)	197 (72.2)	0.958
*yes*	161 (27.9)	85 (28.0)	76 (27.8)	

^a^ The percentages were calculated excluding missing values.

**Table 2:** Univariate logistic regression for outcomes of physical activities.

	Organized Activities (Sports) <3 Days/Week OR (95% CI)	Organized Activities (Sports) <2 Days/Week OR (95% CI)	Non-Organized Activities (Games) <3 Days/Week OR (95% CI)	Physical Activities (Sports + Games) <7 h/Week OR (95% CI)	Car/Bus to Go to School OR (95% CI)	
					Winter	Spring/Autumn
Child’s sex						
Male *						
female	1.6 (1.0–2.5)	1.5 (1.1–2.1)	1.2 (0.8–1.6)	1.4 (0.9–2.0)	0.9 (0.6–1.3)	0.9 (0.7–1.3)
Mother’s nationality						
Italian *						
foreign	2.3 (1.3–4.0)	2.3 (1.6–3.3)	1.3 (0.9–1.8)	0.9 (0.6–1.4)	0.3 (0.2–0.4)	0.3 (0.2–0.4)
Father’s nationality						
Italian *						
foreign	2.9 (1.5–5.6)	2.4 (1.7–3.6)	1.3 (0.9–1.9)	0.9 (0.6–1.4)	0.3 (0.2–0.4)	0.3 (0.2–0.5)
Parents’ nationality						
both Italian *						
at least one foreign	2.0 (1.2–3.5)	2.3 (1.6–3.3)	1.3 (0.9–1.9)	1.0 (0.7–1.5)	0.3 (0.2–0.5)	0.3 (0.2–0.5)
Mother’s education						
<high school *						
high school	0.4 (0.2–0.9)	0.6 (0.4–0.9)	0.9 (0.6–1.4)	0.9 (0.5–1.6)	2.0 (1.2–3.2)	3.0 (1.8–4.9)
university	0.3 (0.1–0.6)	0.2 (0.1–0.4)	0.8 (0.5–1.3)	0.9 (0.5–1.6)	2.2 (1.4–3.6)	2.9 (1.8–4.8)
Father’s education						
<high school *						
high school	0.8 (0.4–1.5)	0.6 (0.4–0.9)	1.3 (0.8–1.9)	1.6 (1.0–2.6)	1.4 (0.9–2.2)	1.5 (1.0–2.2)
university	0.4 (0.2–0.8)	0.3 (0.2–0.5)	1.4 (0.9–2.3)	1.8 (1.1–3.1)	1.3 (0.8–2.2)	1.4 (0.9–2.2)
Parents’ education						
<high school (both parents) *						
high school (at least one parent)	0.7 (0.3–1.7)	0.9 (0.5–1.5)	0.8 (0.5–1.3)	0.9 (0.5–1.6)	2.0 (1.1–3.4)	3.4 (1.9–6.2)
university (only one parent)	0.3 (0.1–0.8)	0.4 (0.2–0.7)	1.0 (0.5–1.7)	1.1 (0.5–2.1)	2.1 (1.2–3.8)	2.4 (1.3–4.6)
university (both parents)	0.3 (0.1–0.8)	0.2 (0.1–0.4)	0.9 (0.5–1.6)	1.0 (0.5–2.1)	2.4 (1.2–4.5)	3.7 (1.9–7.1)
Mother’s weight status						
under/normal weight *						
overweight/obese	1.5 (0.9–2.6)	1.2 (0.8–1.7)	1.3 (0.9–1.9)	1.1 (0.7–1.7)	0.5 (0.3–0.7)	0.7 (0.5–1.0)
Father’s weight status						
normal weight *						
overweight/obese	0.9 (0.6–1.5)	1.3 (0.9–1.9)	1.3 (0.9–1.8)	1.3 (0.9–2.0)	0.9 (0.6–1.3)	0.8 (0.6–1.2)
Parents’ weight status						
both under/normal weight *						
at least one overweight/obese	1.2 (0.7–2.0)	1.4 (1.0–2.0)	1.4 (1.0–2.0)	1.2 (0.8–1.8)	0.8 (0.5–1.3)	0.8 (0.5–1.1)

* Reference category.

**Table 3:** Univariate logistic regression for outcomes of sedentary behaviors.

	Tablet/PC/Mobile Phone Use ≥2 h Daily OR (95% CI)	TV Viewing ≥2 h Daily OR (95% CI)	Sedentary Activities (TV + Tablet/PC/etc) ≥2 h Daily OR (95% CI)	TV in Child’s Bedroom OR (95% CI)
Child’s sex				
Male *				
female	0.7 (0.5–1.1)	0.8 (0.6–1.1)	0.7 (0.5–1.0)	1.0 (0.7–1.4)
Mother’s nationality				
Italian *				
foreign	1.5 (0.9–2.3)	1.2 (0.8–1.6)	1.3 (0.9–1.8)	1.1 (0.8–1.6)
Father’s nationality				
Italian *				
foreign	1.5 (1.0–2.4)	1.4 (1.0–2.1)	1.5 (1.0–2.2)	1.1 (0.7–1.7)
Parents’nationality				
both Italian *				
at least one foreign	1.4 (0.9–2.1)	1.1 (0.8–1.6)	1.2 (0.8–1.7)	1.2 (0.8–1.7)
Mother’s education				
<high school *				
high school	0.4 (0.3–0.7)	0.6 (0.4–1.0)	0.5 (0.3–0.9)	0.6 (0.4–1.0)
university	0.5 (0.3–0.8)	0.4 (0.3–0.7)	0.4 (0.2–0.7)	0.3 (0.2–0.6)
Father’s education				
<high school *				
high school	0.6 (0.4–0.9)	1.0 (0.7–1.5)	1.0 (0.6–1.5)	0.8 (0.5–1.2)
university	0.4 (0.2–0.8)	0.6 (0.4–0.9)	0.5 (0.3–0.8)	0.3 (0.2–0.6)
Parents’ education				
<high school (both parents) *				
high school (at least one parent)	0.4 (0.2–0.8)	0.7 (0.4–1.2)	0.6 (0.3–1.1)	0.6 (0.3–1.0)
university (only one parent)	0.5 (0.2–0.9)	0.5 (0.3–0.8)	0.5 (0.2–0.9)	0.3 (0.1–0.5)
university (both parents)	0.3 (0.2–0.7)	0.3 (0.2–0.6)	0.3 (0.1–0.5)	0.3 (0.1–0.5)
Mother’s weight status				
under/normal weight *				
overweight/obese	1.3 (0.8–2.0)	1.1 (0.7–1.5)	1.4 (1.0–2.1)	1.2 (0.8–1.8)
Father’s weight status				
normal weight *				
overweight/obese	1.8 (1.1–2.9)	1.4 (1.0–2.0)	1.6 (1.1–2.3)	0.8 (0.6–1.3)
Parents’ weight status				
both under/normal weight *				
at least one overweight/obese	1.6 (0.9–2.6)	1.3 (0.9–1.9)	1.5 (1.0–2.1)	1.2 (0.8–1.9)

* Reference category.

**Table 4:** Child’s activities and overweight/obese outcome: univariate and multivariate logistic regression models.

	Univariate	Multivariate *
Child’s Activities	OR (95% CI)	OR (95% CI)
**Physical activities**		
Organized activities (sports)		
≥3 days per week **		
<3 days per week	1.0 (0.6–1.6)	0.7 (0.4–1.3)
Organized activities (sports)		
≥2 days per week **		
<2 days per week	0.9 (0.6–1.3)	0.6 (0.4–1.0)
Non-organized activities (games)		
≥3 days per week **		
<3 days per week	1.2 (0.8–1.7)	1.3 (0.8–1.9)
Physical activities (sports + games)		
≥7 h per week **		
<7 h per week	1.2 (0.8–1.9)	1.2 (0.8–2.0)
How child go to school by season		
winter		
bike/walk **		
motor vehicle (car/bus)	0.8 (0.5–1.1)	1.0 (0.6–1.6)
spring/autumn		
bike/walk **		
motor vehicle (car/bus)	0.8 (0.5–1.2)	0.9 (0.6–1.4)
**Sedentary behaviours**		
Tablet. PC and mobile phone use		
<2 h daily **		
≥2 h daily	2.3 (1.5–3.6)	2.2 (1.4–3.6)
TV viewing		
<2 h daily **		
≥2 h daily	1.3 (0.9–1.9)	1.1 (0.7–1.6)
Sedentary activities (TV + tablet/PC/mobile phone)		
<2 h daily **		
≥2 h daily	1.7 (1.1–2.5)	1.4 (0.9–2.2)
TV in child’s bedroom		
no **		
yes	1.2 (0.8–1.8)	1.1 (0.7–1.7)

* Adjusted for socio-demographic characteristics (parents’ nationality, parents’ education). ** Reference category.

**Table 5:** Univariate logistic regression for dietary habits outcomes.

	Skipping Breakfast OR (95% CI)	Skipping Mid-Morning Snack OR (95% CI)	Fruits/Vegetables <Once a Day OR (95% CI)	Carbonated Drinks ≥Once a Day OR (95% CI)	Sugary Drinks ≥Once a Day OR (95% CI)
**Physical activities**					
Organized activities (sports)					
≥3 days per week *					
<3 days per week	1.2 (0.6–2.6)	1.6 (0.6–4.7)	0.9 (0.5–1.5)	1.7 (0.5–5.8)	1.5 (0.9–2.4)
Organized activities (sports)					
≥2 days per week *					
<2 days per week	1.0 (0.6–1.8)	0.5 (0.2–1.0)	1.1 (0.7–1.7)	1.5 (0.7–3.2)	1.3 (0.9–1.8)
Non-organized activities (games)					
≥3 days per week *					
<3 days per week	1.1 (0.6–1.9)	0.9 (0.5–1.8)	1.4 (0.9–2.1)	1.1 (0.5–2.3)	0.9 (0.7–1.3)
Physical activities (sports + games)					
≥7 h per week *					
<7 h per week	1.0 (0.6–2.0)	0.6 (0.3–1.2)	0.9 (0.6–1.6)	0.9 (0.4–2.0)	0.9 (0.6–1.3)
**Sedentary behaviours**					
Tablet. PC and mobile phone use					
<2 h daily *					
≥2 h daily	3.2 (1.8–5.7)	0.8 (0.3–2.0)	2.0 (1.2–3.3)	2.7 (1.3–5.9)	1.6 (1.0–2.4)
TV viewing					
<2 h daily *					
≥2 h daily	1.5 (0.9–2.5)	1.3 (0.7–2.5)	1.6 (1.0–2.5)	1.4 (0.7–2.9)	1.6 (1.2–2.3)
Sedentary activities (TV + tablet/PC/mobile phone)					
<2 h daily *					
≥2 h daily	1.7 (0.9–3.0)	1.5 (0.7–3.0)	1.9 (1.2–3.2)	1.7 (0.7–3.8)	1.7 (1.2–2.4)
TV in child’s bedroom					
no *					
yes	2.2 (1.3–3.9)	0.9 (0.4–1.9)	1.4 (0.9–2.3)	2.7 (1.3–5.7)	1.5 (1.0–2.1)

* Reference category.

## Supplementary Materials

The following is available online at https://www.mdpi.com/1660-4601/18/6/3221/s1, Table S1: sample characteristics (n.588). Figure S1: physical activities (A) and sedentary behaviors (B) by child’s sex. Males (*n* = 313) − Females (*n* = 275).
