# Supplementary material for: Physical and Sedentary Activities and Childhood Overweight/Obesity: A Cross-Sectional Study among First-Year Children of Primary Schools in Modena, Italy
Source: Int J Environ Res Public Health. 2021 Mar 20;18(6):3221. doi: 10.3390/ijerph18063221 (PMC8003752; doi:10.3390/ijerph18063221)
Supplement: Supplementary file 1 [file ijerph-18-03221-s001.pdf]

Table S1. Sample characteristics (n.588).

**Sample characteristics**	**n (%)[a]**
Child's sex	
male	313 (53.2)
female	275 (46.8)
Child's weight status	
under/normal weight	440 (74.8)
overweight/obese	148 (25.2)
Mother's nationality	
Italian	383 (67.0)
foreign	189 (33.0)
Father's nationality	
Italian	405 (73.4)
foreign	147 (26.6)
Parents'nationality	
both Italian	371 (64.9)
at least one foreign	201 (35.1)
Mother's education	
<high school	114 (19.6)
high school	255 (43.8)
university	213 (36.6)
Father's education	
<high school	145 (25.9)
high school	270 (48.2)
university	145 (25.9)
Parents' education	
<high school (both parents)*	76 (13.6)
high school (at least one parent)	234 (41.9)
university (only one parent)	145 (25.9)
university (both parents)	104 (18.6)
Mother's weight status	
under/normal weight	366 (65.8)
overweight/obese	190 (34.2)
Father's weight status	
normal weight	211 (42.2)
overweight/obese	289 (57.8)
Parents' weight status	
both under/normal weight	157 (30.0)
at least one overweight/obese	366 (70.0)

[a]The percentages were calculated excluding missing values.

A:

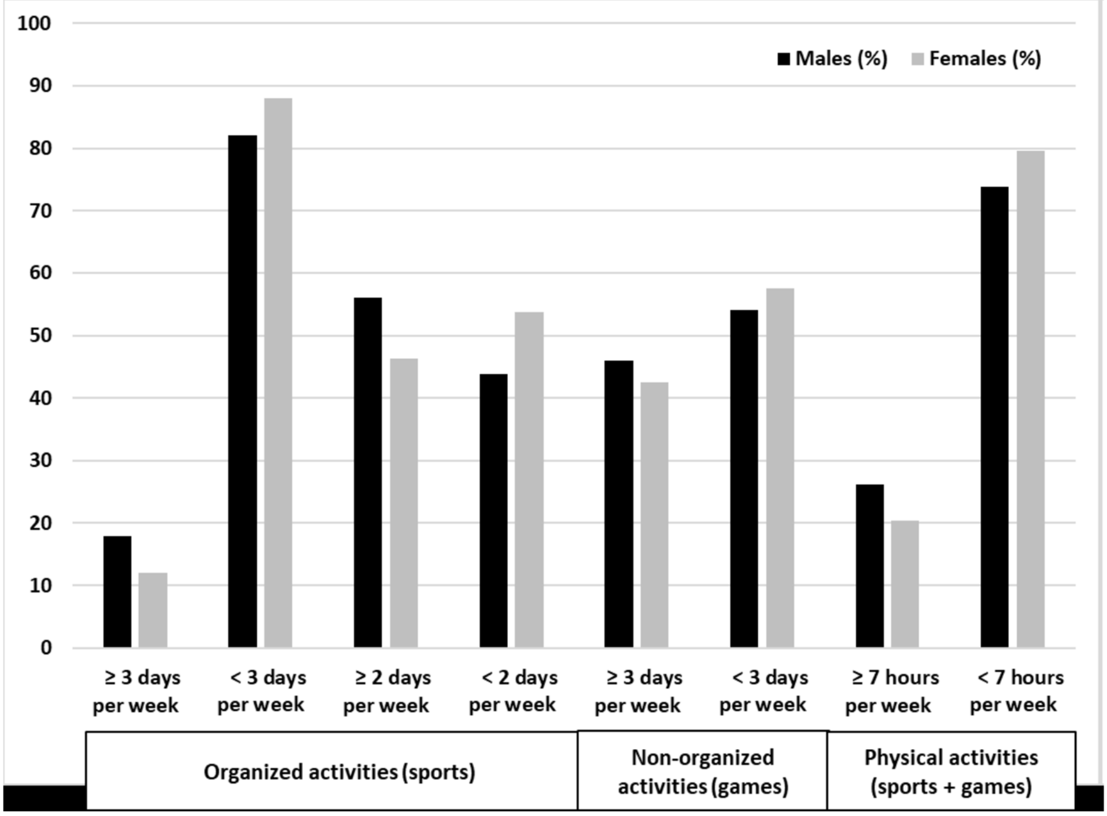

B:

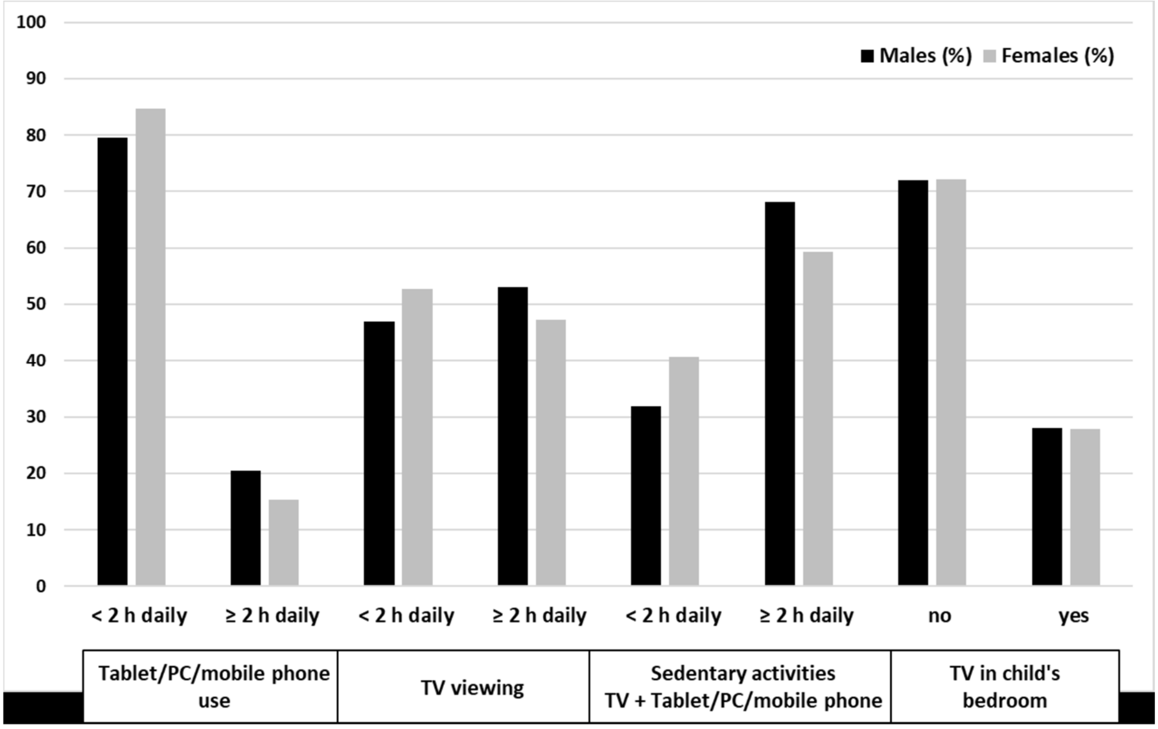

Figure S1: Physical activities (A) and sedentary behaviors (B) by child's sex. Males (n=313) - Females (n=275).
